# Methylation Heterogeneity and Gene Expression of SPG20 in Solid Tumors

**DOI:** 10.3390/genes13050861

**Published:** 2022-05-12

**Authors:** Vincenza Ylenia Cusenza, Luca Braglia, Raffaele Frazzi

**Affiliations:** 1 Laboratory of Translational Research, Azienda Unità Sanitaria Locale—IRCCS di Reggio Emilia, 42123 Reggio Emilia, Italy; vincenzaylenia.cusenza@ausl.re.it; 2 Research and Statistics Infrastructure, Azienda Unità Sanitaria Locale—IRCCS di Reggio Emilia, 42123 Reggio Emilia, Italy; luca.braglia@ausl.re.it

**Keywords:** *SPG20*, DNA methylation, gene expression regulation

## Abstract

Introduction. The downregulation of the Spastic Paraplegia-20 (*SPG20*) gene is correlated with a rare autosomal recessive disorder called Troyer Syndrome. Only in recent years has *SPG20* been studied and partially characterized in cancer. *SPG20* has been shown to be hypermethylated in colorectal cancer, gastric cancer, non-Hodgkin’s lymphoma and hepatocellular carcinoma. In this study, we analyze the methylation status and the gene expression of *SPG20* in different tumors of various histological origins. Methods. We analyzed the data generated through Infinium Human Methylation 450 BeadChip arrays and RNA-seq approaches extrapolated from The Cancer Genome Atlas (TCGA) database. The statistics were performed with R 4.0.4. Results. We aimed to assess whether the hypermethylation of this target gene was a common characteristic among different tumors and if there was a correlation between the m-values and the gene expression in paired tumor versus solid tissue normal. Overall, our analysis highlighted that *SPG20* open sea upstream the TSS is altogether hypermethylated, and the tumor tissues display a higher methylation heterogeneity compared to the solid tissue normal. The gene expression evidences a reproducible, higher gene expression in normal tissues. Conclusion. Our research, based on data mining from TCGA, evidences that colon and liver tumors display a consistent methylation heterogeneity compared to their normal counterparts. This parallels a downregulation of *SPG20* gene expression in tumor samples and suggests a role for this multifunctional protein in the control of tumor progression.

## 1. Introduction

DNA methylation dysregulation plays a role in tumorigenesis and can be used as a biomarker in oncology [1]. Several genes have emerged over the years to be epigenetically regulated in different cancers; among these, there is Spastic paraplegia-20 (*SPG20*). *SPG20*, also known as *SPART*, is the gene that encodes the spartin protein. *SPG20* is known to be involved in Troyer syndrome, where its mutation leads to the downregulation of spartin expression [2,3,4]. Spartin plays several roles [5]. It has been shown to participate in the transport of Epidermal Growth Factor Receptor (EGFR) [6], in the energy and metabolism processes of mitochondria and in the metabolism of lipid droplets [7,8,9,10,11,12,13]. It has also been reported that spartin plays a role as the regulator of cytokinesis in the cell cycle and microtubule stability [5,14,15,16]. However, there is little information available regarding this gene in different types of tumors. Very limited evidence has been published reporting mutations in the *SPG20* locus in cancer. Specifically, 7% of a cohort of 149 esophageal cancer analyzed by whole-exome sequencing bore mutations [17]. In chronic lymphocytic leukemia, *SPG20* emerged as differentially expressed with regards to the *IGVH* mutational status [18]. In this study, though, no specific mutations were described and no mechanistic evidence reported. Overall, no direct connection between *SPG20* mutations and cancer predisposition has been confirmed thus far.

It has been demonstrated that, in colorectal cancer, hepatocellular carcinoma, gastric cancer and non-Hodgkin’s lymphoma (NHL), *SPG20* is epigenetically regulated [19,20,21,22,23]. In all these cancers, the *SPG20* promoter results hypermethylated, and its hypermethylation correlates negatively with its expression. We decided to address the question of whether the epigenetic regulation of *SPG20* is a generalized phenomenon common to cancers of different histological origin and whether there is a correlation with the expression levels. We analyzed the data of the Illumina 450 methylation BeadChip arrays available in The Cancer Genome Atlas (TCGA) database for several cancer types, specifically the open sea region located immediately upstream of the transcription starting site (TSS), and verifying if there were differences between the cancer and normal tissues. In this analysis, we chose the datasets where the methylation data of the normal counterpart and the gene expression data were also available in the TCGA. By adopting these filters, we could analyze six different human solid tumors: bladder, colon, kidney, liver, lung and prostate.

## 2. Materials and Methods

### 2.1. Data Mining of Quantitative Methylation from The Cancer Genome Atlas (TCGA) Database

**Figure 1:**
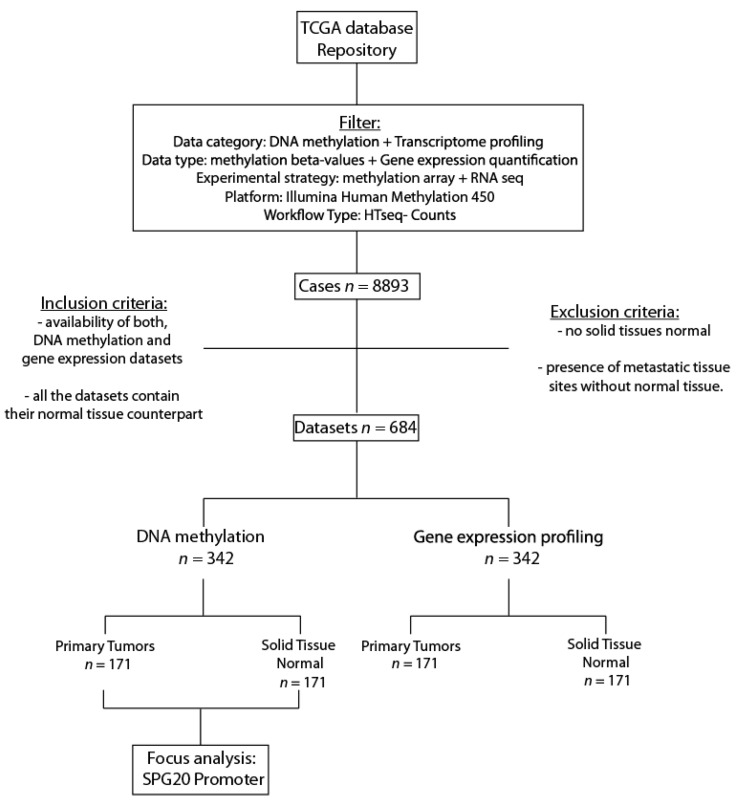
Algorithm of the workflow applied and described in the present research on The Cancer Genome Atlas (TCGA). The inclusion and exclusion criteria are specified.

We used the DNA methylation data (16–46 independent datasets) belonging to 6 different tumor sites. Each tumor site had its solid tissue normal datasets deposited in TCGA (Figure 1). Their DNA methylation profiles were measured experimentally using the Illumina Infinium Human Methylation 450K platform. The analysis focused on the promoter of the target gene: *SPG20*. The data mining for our analysis was performed on TCGA by inserting specific filters: DNA methylation (data category) + methylation β values (data type) + methylation array (experimental strategy) + Illumina Human Methylation 450 (platform).

### 2.2. Data Mining of Gene Expression from The Cancer Genome Atlas (TCGA) Database

We used the gene expression data from the datasets extrapolated for the DNA methylation analysis. The gene expression was measured using RNA-sequencing. The data mining for the analysis was performed on TCGA by inserting the following filters: DNA methylation and transcriptome profiling (data category) + methylation β-values and gene expression quantification (data type) + HTseq-Counts (workflow type).

### 2.3. Inclusion Criteria

All the selected data met the following criteria: (1) availability of the datasets of genome-wide methylation by the Illumina Infinium Human Methylation 450K platform, (2) availability of the gene expression datasets by RNA-sequencing and (3) availability of solid tissue normal counterpart in the datasets (Figure 1).

### 2.4. Exclusion Criteria

The exclusion criteria were: (1) no solid tissue normal available and the (2) presence of metastatic tissue sites without normal tissue.

### 2.5. Statistical Analysis

A descriptive analysis of the dataset was based on the median and IQR for both m-values and HTseq values. Distribution of the HTseq values was compared between normal and tumoral specimens using the Wilcoxon paired rank sum test. Analysis of the probes dataset was based on m-values instead of β-values (obtained from the latter by log_2_[(β)/(1 − β)], in order to reduce the skewness and improve the normality) and was aimed at evaluating the overall differences between means in tumor specimens versus normal ones. It was performed, for each organ, estimating a linear mixed model with an m-value as the dependent variable and group (Normal/Tumor) as the main fixed effect; we added two crossing random effects, (patient and probe), as needed by design, and modeled the heteroscedasticity associated with the group (Normal/Tumor), allowing different error variances to be estimated for each stratum. For each estimate, we reported the group coefficient (indicated the delta associated with tumor specimens compared to the normal ones), accompanied by the confidence interval and *p*-value, according to the Wald test. Confidence intervals were two-tailed and calculated considering a 0.95 confidence level; performed tests were considered statistically significant when the *p*-values were < 0.05. Statistical analysis was performed using the R 4.0.4 R Core Team [24].

## 3. Results

The search yielded a total of 684 datasets divided into tumor tissues and solid tissue normal. The 171 tumor tissues encompassed six different organs, collecting a variable number of datasets for each site. The solid tissue normals were unevenly subdivided between the different tumors and only the ones paired with their tumor counterpart in each specific cohort were chosen (Figure 1; the datasets are listed in the “Supplementary File S1”).

Our interest focused on the methylation status of the *SPG20* promoter, since it emerges consistently hypermethylated in some cancers. Nine probes mapping close and upstream of the TSS of the gene *SPG20* (NC_000013.11) have been previously identified and analyzed [20]. They map into an open sea region (located >4 kb away from the noted CpG island) characterized by high β-values that we recently described (human methylation 450 BeadChip on 28 DLBCL cell lines). The open sea lies upstream of the *SPG20* TSS (transcript variant 4). Since this open sea region does not map within a known annotated CpG island, we aimed at characterizing this specific region for which there are no available data yet on solid cancers.

The nine probes are:

cg09190748

cg15754752

cg21056788

cg21484515

cg05635923

cg00084432

cg13486491

cg09410612

cg14410132

Two of the probes previously reported in 28 lymphoma cell lines (cg13486491 and cg09410612) did not yield any data with the present research criteria. In the last part of the research, we assessed the quantitative methylation of the annotated CpG island located across exon 1 in colon and hepatocellular carcinomas. This island is recognized by 15 probes (human methylation 450 BeadChip), and the results are described in Section 3.7.

### 3.1. Bladder Carcinoma

**Figure 2:**
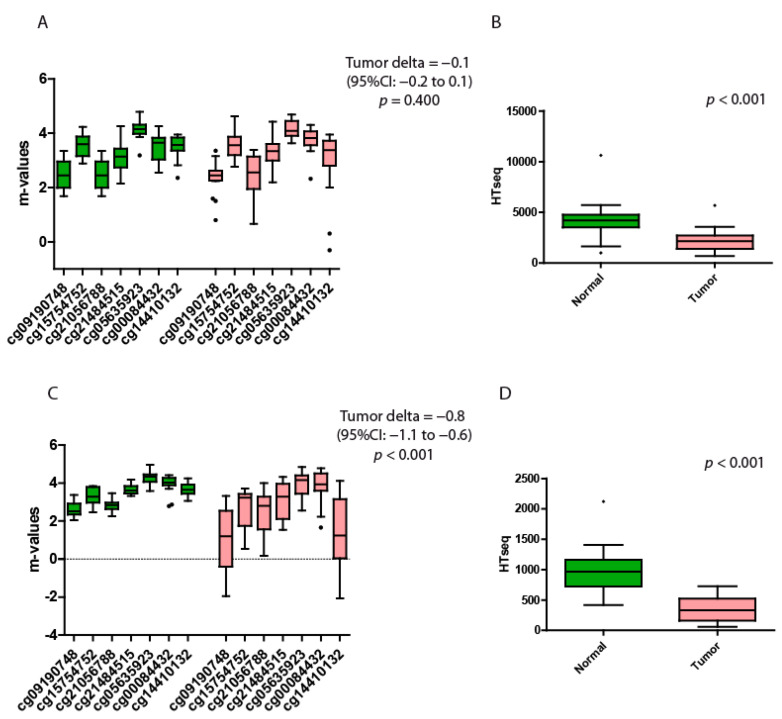
Quantitative methylation and gene expression analysis of bladder and colorectal datasets. (**A**) Bladder m-values of the 7 probes located at the open sea region of the locus SPG20. (**B**) Bladder expression levels of SPG20 in normal versus tumor tissues. The *p*-value indicates the statistical significance of the data. (**C**) Colorectal m-values of the 7 probes located at the open sea region of the locus SPG20. (**D**) Colorectal expression levels of SPG20 in normal versus tumor tissues. The *p*-value indicates the significance of the data. Green boxplots represent the nontumoral tissues (solid tissue normal datasets), while red boxplots represent the tumor tissues. The black dots represent the outliers. Each probe is identified with a specific alphanumeric code. The tumor delta is a value that represents the variation between the two groups. The higher the value of tumor delta, the greater the variation between the two groups. Each box plot displays the median value, and the whiskers are the interval between the 1st and 3rd quartiles.

We collected and analyzed 16 pairs of normal/tumor datasets. The promoter region of *SPG20* in this type of cancer is characterized by high β-values in the primary tumors but also in solid tissue normal. The β-values range from 0.76 to 0.97 in solid tissue normal and from 0.45 to 0.96 in tumor samples, demonstrating a higher heterogeneity in the latter. The m-values do not evidence any significant differences between normal and tumor tissues (Table 1 and Figure 2A).

The gene expression analysis consistently shows that the expression (HTseq) is considerably higher in solid tissue normals than in their respective tumors (*p* < 0.001 by the Wilcoxon paired rank sum test; Figure 2B).

### 3.2. Colorectal Carcinoma

In this site, we collected and analyzed 16 pairs of normal/tumor datasets. The heterogeneity of the β-values in the cancer samples was very high, from 0.19 to 0.97. Conversely, the β-values of the normal tissues were homogeneous, ranging from 0.81 to 0.97. The difference between the m-values of these two groups was highly significant (Table 2 and Figure 2C). The tumor tissues had an average m-values almost one point lower than their normal counterparts (Tumor delta = −0.8).

The gene expression data showed a consistently higher expression of spartin in solid tissue normal compared to the tumor tissues (*p* < 0.001 by the Wilcoxon paired rank sum test; Figure 2D).

### 3.3. Kidney Carcinoma

**Figure 3:**
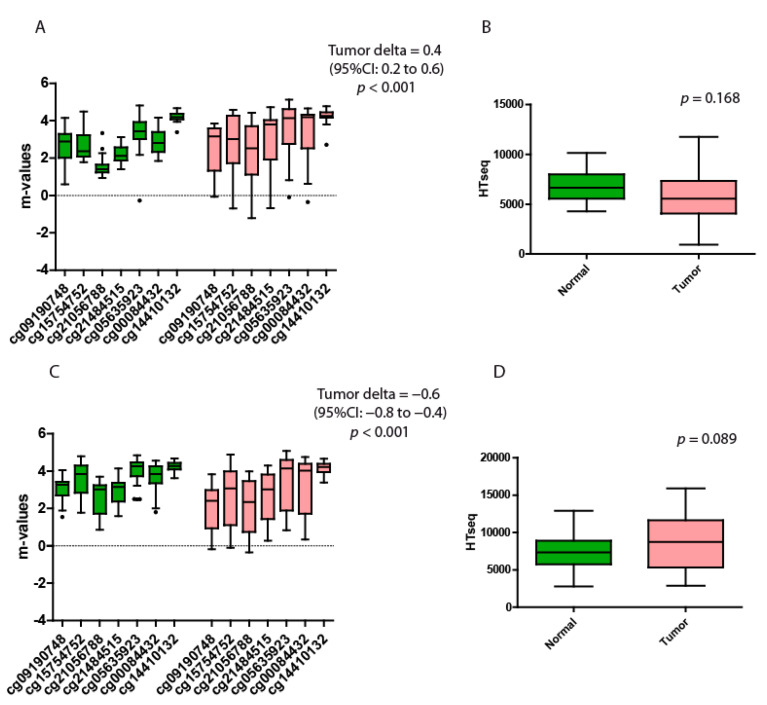
Quantitative methylation and gene expression analysis of the KIRP and KIRC datasets. (**A**) KIRP m-values of the 7 probes located at the open sea region of the locus SPG20. (**B**) KIRP expression levels of SPG20 in normal versus tumor tissues. The *p*-value indicates the statistical significance of the data. (**C**) KIRC m-values of the 7 probes located at the open sea region of the locus SPG20. (**D**) KIRC expression levels of SPG20 in normal versus tumor tissues. The *p*-value indicates the significance of the data. Green boxplots represent the nontumoral tissues (solid tissue normal datasets), while red boxplots represent the tumor tissues. The black dots represent the outliers. Each probe is identified with a specific alphanumeric code. The tumor delta is a value that represents the variation between the two groups. The higher the value of the tumor delta, the greater the variation between the two groups. Each box plot displays the median value and the whiskers the interval between the 1st and 3rd quartiles.

A total of 46 pairs of normal/tumor datasets were collected. The samples were divided according to the two most frequent subtypes: kidney renal papillary cell carcinoma (KIRP; 22 pairs) and kidney renal clear cell carcinoma (KIRC; 24 pairs). The KIRP β-values ranged from 0.30 to 0.97, whereas, in normal tissues, ranged from 0.45 to 0.97. The KIRC tumor β-values ranged from 0.44 to 0.97, whereas, in normal tissues, ranged from 0.65 to 0.97. Both papillary cell carcinoma and clear cell carcinoma displayed highly significant differences between the tumor and their normal counterparts (Table 3 and Table 4 and Figure 3A,C, respectively).

However, the differences between the two subtypes emerged at the level of gene expression. The KIRP tumor samples displayed a lower spartin expression compared to their normal counterparts, whereas the KIRC tumor samples displayed higher levels compared to their normal counterparts. None of these differences were significant (Figure 3B,D).

### 3.4. Hepatocellular Carcinoma

**Figure 4:**
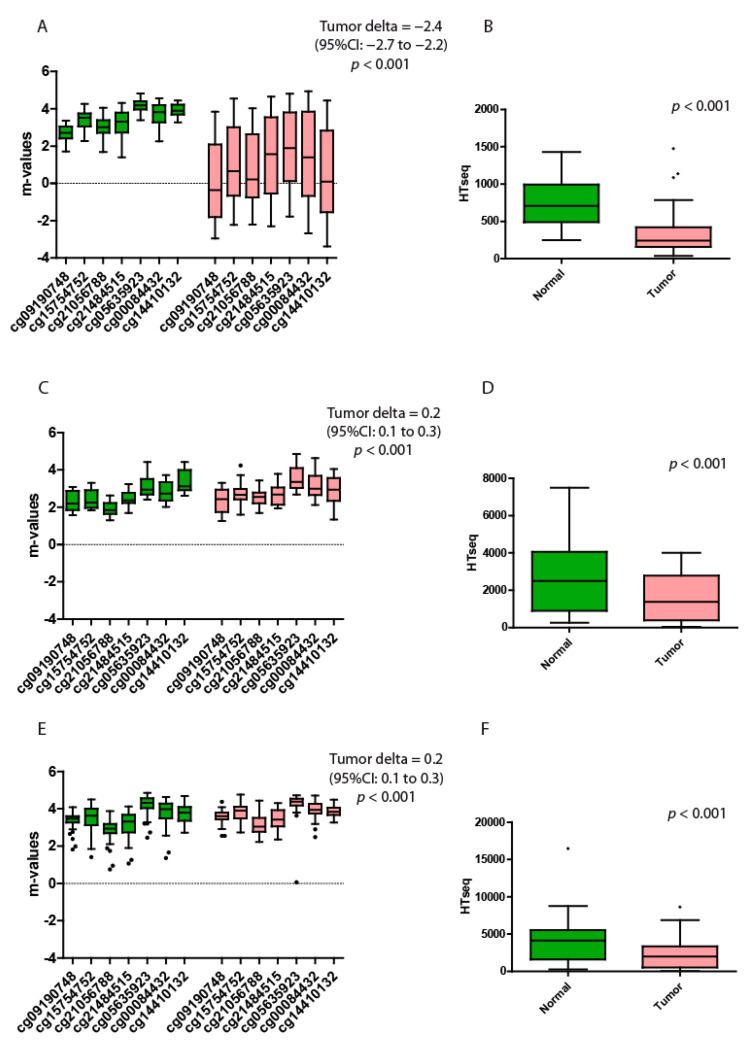
Quantitative methylation and gene expression analysis of hepatocellular, lung and prostate carcinomas. (**A**) Hepatocellular m-values of the 7 probes located at the open sea region of the locus SPG20. (**B**) Hepatocellular expression levels of SPG20 in normal versus tumor tissues. The *p*-value indicates the statistical significance of the data. (**C**) Lung m-values of the 7 probes located at the open sea region of the locus SPG20. (**D**) Lung expression levels of SPG20 in normal versus tumor tissues. The *p*-value indicates the significance of the data. (**E**) Prostate m-values of the 7 probes located at the open sea region of the locus SPG20. (**F**) Prostate expression levels of SPG20 in normal versus tumor tissues. Green boxplots represent the nontumoral tissues (solid tissue normal datasets), while red boxplots represent the tumor tissues. The black dots represent the outliers. Each probe is identified with a specific alphanumeric code. The tumor delta is a value that represents the variations between the two groups. The higher the value of the tumor delta, the greater the variations between the two groups. Each box plot displays the median value and the whiskers the interval between the 1st and 3rd quartiles.

A total of 41 tumor samples and solid tissue normal were collected and analyzed. The β-values of the target gene were from 0.09 to 0.97 in hepatocellular carcinoma. The normal tissues were instead from 0.73 to 0.97. The differences between the m-values of the two groups were highly significant. In liver, the tumor delta highlights the major variations, and the tumor tissues have, on average, m-values 2.4 points lower than the nontumor samples (Table 5 and Figure 4A).

The gene expression data showed a significantly higher expression of spartin in the normal counterparts when compared to the tumor samples (*p* < 0.001 by the Wilcoxon paired rank sum test; Figure 4B).

### 3.5. Lung Carcinoma

At variances with the other tumor types, lung carcinoma showed more homogeneous methylation profiles in both normal and tumor tissues (*n* = 19). Despite the fact that the β-values ranged from 0.71 to 0.97 in cancer tissues and from 0.71 to 0.96 in normal tissues, these differences were statistically different (Table 6 and Figure 4C).

The gene expression data confirmed the previous observations, since solid tissue normal expressed significantly higher spartin compared to their tumor counterpart (*p* < 0.001 by the Wilcoxon paired rank sum test; Figure 4D).

### 3.6. Prostate Carcinoma

*SPG20* in prostate tissues (*n* = 33) was stably methylated in both tumor and normal tissues, with β-values ranging from 0.51 to 0.96 in cancer tissues and from 0.63 to 0.97 in normal tissues. These differences were statistically different but unlikely to bear any biological relevance (Table 7 and Figure 4E). The gene expression data demonstrated, again, that the nontumor prostate expressed more spartin than the tumor counterpart, and this difference was statistically different (*p* < 0.001 by the Wilcoxon paired rank sum test; Figure 4F).

### 3.7. CpG Island Analysis in Colon and Hepatocellular Carcinoma

**Figure 5:**
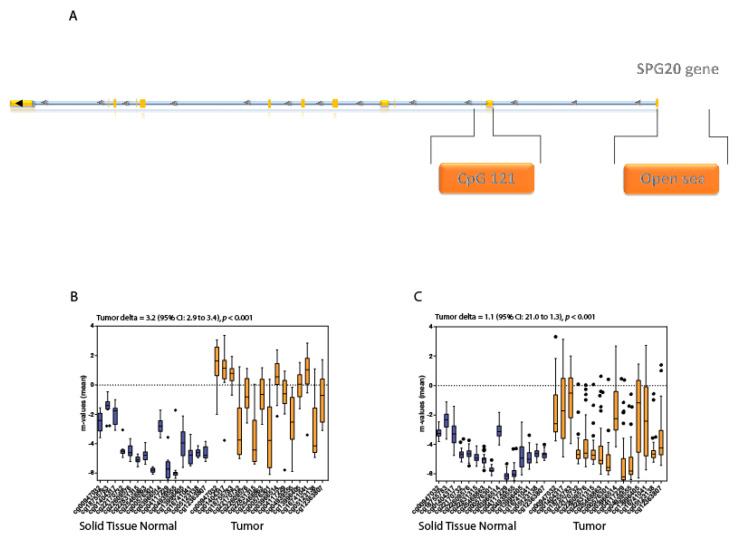
(**A**) Schematic representation of the SPG20 gene with the localization of the two analyzed regions: CpG_121 and open sea. (**B**,**C**) m-values analysis of the methylation status of the canonical CpG island (CpG_121) in colorectal and hepatocellular carcinoma. Blue boxplots represent nontumoral tissues, while orange boxplots represent the tumor tissues. The tumor delta represents the variations between the two groups; the *p*-value indicates the statistical significance of the data. Each box plot displays the median value and the whiskers the interval between the 1st and 3rd quartiles.

We selected the tumor sites displaying the higher tumor delta values for further analysis aimed at assessing the distribution of the m-values of the probes within the CpG island of the gene body (CpG_121, according to the iMETHYL integrative DNA methylation database [25]). These were colon and hepatocellular carcinomas (Figure 5A). We analyzed a total of 15 probes for each normal and tumor paired dataset (*n* = 114). The m-values are represented in Figure 5B. Additionally, in this intracellular CpG island, the differences in each tumor site were statistically significant (*p* < 0.001). Wide tumor delta values existed in between the solid tissue normal and tumor datasets (tumor delta of 3.2 and 1.1 for the colon and liver, respectively), with the tumor samples displaying consistently higher m-values (Figure 5B,C). These data supported the biological evidence that *SPG20* mRNA was lower in all tumor samples, strengthening the hypothesis of epigenetic regulation.

## 4. Discussion

The aberrant methylation in the promoters of oncosuppressors or oncogenes in cancer has been confirmed by several authors over the years and could be a prognostic biomarker in some settings [26,27,28,29,30]. In the manuscript by He L. and coworkers, the methylation differences of the *SPG20* promoter between cancerous (*n* = 160 HCC) and adjacent benign liver tissues were confirmed [21]. In the same work, the expression of *SPG20* in different HCC cell lines was evaluated, demonstrating that *SPG20* was downregulated when compared with the normal hepatocytes. The methylation of *SPG20* was also correlated to multi-satellite tumors and metastasis [21].

The recent manuscript by Wei K.L. and coworkers reported the data obtained through an Illumina 850K methylation microarray in AGS gastric cancer cell lines and in cells depleted of STAT3 [22]. *SPG20* was identified as a putative STAT3 epigenetic target, and the promoter of this target gene was hypomethylated in STAT3-depleted AGS cells. The methylation analysis of *SPG20* by pyrosequencing in a cohort of gastritis, intestinal metaplasia and paired gastric cancer patient samples showed that a higher methylation percentage was observed in gastric tumors, intermediate in intestinal metaplasia and low in adjacent normal and gastritis tissues. These data were confirmed by the analysis from two publicly available databases (GSE103186 and TCGA) [22]. At variances with the data that we recently described and characterized in NHL cell lines [20], the expression of *SPG20* could be reactivated by decitabine treatment in the gastric cell lines. Thus, differences among cell lines of different histological origin are plausible and cannot be excluded.

Colorectal carcinoma, adenomas and normal mucosa have been analyzed by quantitative methylation-specific PCR (qMSP) and confirmed by direct bisulfite sequencing. Additionally, in this setting, *SPG20* was found to be methylated in 91% of carcinomas, 75% of adenomas and 2% of normal mucosa, and the results were also reproduced in a validation cohort. Collectively, these results showed that *SPG20* promoter is hypermethylated in the majority of colorectal carcinomas and adenomas but very rarely in normal epithelium [23]. This evidence led to the development of early screening indicators for colorectal cancer, including the methylation of *SPG20* [31,32,33,34].

In another article published in 2018, it was shown that the hypermethylation of *SPG20* could be used as a biomarker for gastric cancer screening and that the absence of the spartin expression could be used as a prognostic factor for gastric cancer patients [35]. The knockout of *SPG20* promotes gastric cancer cell proliferation, in vitro G2/M arrest and in vivo tumor growth through the activation of the EGFR/MAPK pathway. The patients with low levels of *SPG20* expression exhibited a worse prognosis compared with the patients with a higher expression. Among these patients, 56.7% exhibited *SPG20* hyper-methylation [35].

*SPG20* was also found to be hypermethylated in follicular lymphoma (FL) and Diffuse Large B Cell Lymphoma (DLBCL) when compared to follicular hyperplasia [19]. Furthermore, spartin is expressed in peripheral blood mononuclear cells from donors and inversely correlates with the degree of methylation in its promoter [20].

In the present manuscript, we analyzed several datasets of six solid tumors: bladder, colon, kidney, liver, lung and prostate cancers. We focused on a specific region upstream of the TSS that we previously characterized through the Infinium 450K methylation array. The bioinformatics analysis located this region in an open sea right upstream the TSS of *SPG20* (GRCh37.p13, pos. 36944294-36945555) in a panel of 28 DLBCL cell lines, and it was hypermethylated in 24 out of 28 of them [20]. Here, we showed that, surprisingly, the solid tissue normal still displayed high β-values. The differences between the tumor samples and the solid tissue normal concerned the heterogeneity of the distribution, which was significantly different: higher for tumors and narrower for normal tissues.

Furthermore, the gene expression analysis of the same samples consistently showed that the tumor samples expressed lower mRNA levels compared to their nontumor counterparts. This was true for all the analyzed tissues except the kidney, where clear cell carcinomas (KIRC) represent separate entities compared to papillary carcinomas (KIRP).

However, while colorectal and hepatocellular carcinoma display significantly different m-values in the analyzed open sea region, the bladder, lung and prostate do not show differences. Still, the HTseq levels are higher in the solid tissue normal when compared to the tumor samples in all these tissues, suggesting that the epigenetic modulation of *SPG20* expression is not entirely defined by the levels of methylation at the open sea region.

Finally, we chose colorectal and hepatocellular carcinomas, because the tumor delta values in their open sea regions were wider, and we also analyzed the CpG_121 island located within the coding region of the gene at the level of exon 1. In this CpG island, the m-values of the tumor samples were significantly higher than the m-values of the solid tissue normal, suggesting that they play a role in the observed downregulation of *SPG20* expression in their respective tumor samples.

## 5. Conclusions

This evidence, collectively, showed that spartin downregulation is a common feature of neoplastic cells of different histological origins. At the same time, this evidence showed that the epigenetic regulation of *SPG20* does not rely entirely on the absolute levels of methylation of the tumor samples. Epigenetically regulated oncosuppressors are attractive therapeutic targets. Knowledge of the actual regulatory mechanisms for *SPG20* is therefore mandatory.

Further experiments aimed at the identification of the CpG-rich sequences responsible for the transcriptional regulation and the identification of which DNA methyltransferase is responsible for the differential methylation are now warranted.

## Figures and Tables

**Table 1:** Bladder β-values and statistics.

	*n*	NA	Min	Max	Median	1st Qu.	3rd Qu.	Mean	Std. Dev.
Normal	112	0	0.7620276	0.9651312	0.91071965	0.87729694	0.93414696	0.89934474	0.04508459
Tumor	112	0	0.44663269	0.9627281	0.9120437	0.86878698	0.93518638	0.88795607	0.08184771
All	224	0	0.44663269	0.9651312	0.91142945	0.87303703	0.93492645	0.89365041	0.06617271

**Table 2:** Colorectal β-values and statistics.

	*n*	NA	Min	Max	Median	1st Qu.	3rd Qu.	Mean	Std. Dev.
Normal	112	0	0.8053819	0.96909	0.92112001	0.88554056	0.94098467	0.91033294	0.03811312
Tumor	112	0	0.1920369	0.9663544	0.9027551	0.7546837	0.93482298	0.81605931	0.17675964
All	224	0	0.1920369	0.96909	0.9110426	0.85669331	0.93951865	0.86319612	0.13603978

**Table 3:** KIRC β-values and statistics.

	*n*	NA	Min	Max	Median	1st Qu.	3rd Qu.	Mean	Std. Dev.
Normal	168	0	0.6456412	0.96643075	0.92073055	0.88218865	0.94753695	0.90297794	0.0622972
Tumor	168	0	0.4394583	0.9712161	0.9114003	0.75248563	0.94633813	0.8407438	0.13942641
All	336	0	0.4394583	0.9712161	0.91860305	0.84688073	0.94671613	0.87186087	0.11223497

**Table 4:** KIRP β-values and statistics.

	*n*	NA	Min	Max	Median	1st Qu.	3rd Qu.	Mean	Std. Dev.
Normal	154	0	0.4536597	0.9655634	0.8696699	0.80179138	0.92289783	0.85250608	0.08696032
Tumor	154	0	0.3016275	0.9721765	0.92967585	0.82169793	0.94986938	0.86169078	0.13880493
All	308	0	0.3016275	0.9721765	0.9010088	0.80486935	0.94558093	0.85709843	0.11572345

**Table 5:** Liver β-values and statistics.

	*n*	NA	Min	Max	Median	1st Qu.	3rd Qu.	Mean	Std. Dev.
Normal	287	0	0.72612426	0.96593354	0.91882459	0.88653807	0.93923236	0.9082021	0.04362828
Tumor	287	0	0.08751658	0.96846142	0.62074025	0.3465958	0.89729661	0.60988361	0.27688223
All	574	0	0.08751658	0.96846142	0.88962457	0.62280146	0.9324279	0.75904286	0.24799667

**Table 6:** Lung β-values and statistics.

	*n*	NA	Min	Max	Median	1st Qu.	3rd Qu.	Mean	Std. Dev.
Normal	133	0	0.71204958	0.95575472	0.85993052	0.81549489	0.89308814	0.85417608	0.05425518
Tumor	133	0	0.70587211	0.96651218	0.87693043	0.84265481	0.90442023	0.86952819	0.05256677
All	266	0	0.70587211	0.96651218	0.86613566	0.82809024	0.89847697	0.86185213	0.05386856

**Table 7:** Prostate β-values and statistics.

	*n*	NA	Min	Max	Median	1st Qu.	3rd Qu.	Mean	Std. Dev.
Normal	231	0	0.62718377	0.96668304	0.92332385	0.89358549	0.94325791	0.9092877	0.05379345
Tumor	231	0	0.51030674	0.96444073	0.9327408	0.91461837	0.94556577	0.92403296	0.03902904
All	462	0	0.51030674	0.96668304	0.92838193	0.90467613	0.94461771	0.91666033	0.04752035

## Data Availability

Not applicable.

## Supplementary Materials

The following supporting information can be downloaded at: https://www.mdpi.com/article/10.3390/genes13050861/s1, File S1: Data availability statement.
