# Supplementary material for: Methylation Heterogeneity and Gene Expression of SPG20 in Solid Tumors
Source: Genes (Basel). 2022 May 12;13(5):861. doi: 10.3390/genes13050861 (PMC9140344; doi:10.3390/genes13050861)
Supplement: Supplementary file 1 [file genes-13-00861-s001.zip › genes-1716602-supplementary.pdf]

Data availability statement

Bladder cases:

TCGA
GC-A3BM
CU-A0YN
CU-A0YR
GC-A3WC
K4-A5RI
K4-A3WV
K4-A54R
BT-A2LA
GD-A3OQ
BT-A20W
GD-A2C5
BT-A20N
BT-A20U
BT-A20R
GC-A6I3
GD-A3OP

Colon cases:

TCGA
AZ-6601
AA-3697
AA-3713
A6-2682
A6-2685
A6-5667
A6-2671
A6-2675
A6-2686
AZ-6599
A6-2680
AZ-6600
AA-3655
AZ-6598
AA-3660
AA-3712

Kidney cases:

KIRC

TCGA
CZ-4865
CZ-4864
CZ-5467
CZ-5465
CZ-5466
CZ-4863
CZ-5469
CZ-5470
CZ-5454
CZ-5463
CZ-5458
B0-5711
CZ-5457
CZ-5461
CZ-5452
CZ-5451
CZ-5453
CZ-5455
CZ-5468
CZ-5462
B0-5402
B0-5712
CZ-5456
B0-4712

KIRP

TCGA
BQ-7046
BQ-5888
BQ-7055
BQ-5891
BQ-7051
GL-6846
DZ-6132
BQ-5894
BQ-5875
BQ-7044
BQ-7061
BQ-5882
BQ-5879
BQ-5887
BQ-7045
BQ-5884
BQ-5877
DZ-6133
BQ-5890
BQ-7059
BQ-5878
DZ-6134

Liver cases:

TCGA			
DD-A39Z	DD-A1EL	BC-A216	BC-A110
DD-A1EE	DD-A113	BD-A2L6	DD-A39X
DD-A3A1	BC-A10Y	DD-A3A2	
BD-A3EP	DD-A118	DD-A114	
FV-A2QR	DD-A11D	BC-A10W	
DD-A1EG	DD-A39W	BC-A10Z	
BC-A10U	DD-A3A3	BC-A10X	
DD-A1EI	DD-A11C	DD-A1EC	
DD-A11A	EP-A26S	DD-A39V	
DD-A119	BC-A10T	FV-A23B	
DD-A1EH	DD-A1EB	DD-A1EJ	
DD-A116	BC-A10R	EP-A12J	
DD-A11B	ES-A2HT	BC-A10Q	

Lung cases:

TCGA
22-5471
22-5472
73-4676
50-5939
50-5935
49-6745
50-5932
50-5933
50-5931
44-6148
50-5930
44-6145
44-6778
38-4632
50-5936
43-6771
22-5491
22-5482
22-5478

Prostate cases:

TCGA	
EJ-7123	EJ-7786
EJ-7784	EJ-7794
G9-6333	CH-5767
G9-6496	EJ-7792
HC-7737	HC-7752
EJ-7125	EJ-7782
EJ-7789	G9-6356
CH-5768	G9-6499
EJ-7328	G9-6365
EJ-7781	HC-7745
G9-6362	G9-6363
G9-6342	CH-5761
HC-7819	EJ-7317
EJ-7327	
CH-5769	
HC-7742	
G9-6384	
EJ-7785	
EJ-7331	
G9-6351	
